# 1828. The Stratification of HIV Viral Suppression Rates and Related Health Outcomes of UNMC HIV Clinic Patients by Local Zip Code in Omaha, NE

**DOI:** 10.1093/ofid/ofad500.1657

**Published:** 2023-11-27

**Authors:** Mark Carter, Nada Fadul, Jennifer Liu, Adati Tarfa, Brent Cordon, Elizabeth Lyden

**Affiliations:** University of Nebraska Medical Center, Omaha, Nebraska; University of Nebraska Medical Center, Omaha, Nebraska; University of Nebraska Medical Center, Omaha, Nebraska; University of Wisconsin Madison, Madison, Wisconsin; University of Nebraska Medical Center, Omaha, Nebraska; University of Nebraska Medical Center, Omaha, Nebraska

## Abstract

**Background:**

Previous research has established associations between self-reported racial/ethnic identity and poorer HIV outcomes; Black patients have disproportionately higher prevalence and poorer viral suppression rates compared to White patients. Expanding upon this research by identifying local Nebraska zip codes with poorer health outcomes will further characterize the concept of “structural racism” which refers to the perpetuation of racial discrimination by reinforcing systems of oppression related to housing, healthcare, and other social determinants of health.

**Methods:**

Within the patient database at the UNMC HIV specialty clinic, 1,261 adult patients 19 years and older with HIV-1 infection were recruited. The analysis was done with 1097 patients who reside in Nebraska and have SVI data available. Demographic and clinical data were obtained through the patient database. Weighted-average SVI calculations were completed for each patient zip code by using the ratio of total addresses in each census tract to total addresses in the zip code. The SVI score range is 0-1, 0 being lowest risk and 1 being highest risk. Areas with SVI scores ≥ 0.9 have been characterized as being particularly high risk. Fisher’s exact test was used to look at the association of sociodemographic characteristics with the SVI (dichotomized < 0.9 vs. ≥ 0.9). A p-value < 0.05 was considered statistically significant.

**Results:**

There is a statistically significant association between poverty level and SVI group (p = 0.0073). Among patients with SVI < 0.9, 48.1% of patients at or below 100% poverty level compared to 60.9% of patients who have SVI ≥ 0.9 (very high risk). A statistically significant association between race and SVI group (p < 0.0001) is seen; the proportion of Black patients is higher in areas with very high SVI (51.3%) compared to areas with SVI < 0.9 (28.0%). There is a statistically significant association between poor viral suppression and SVI group (p = 0.0076). 11.7% of those who reside in areas with SVI ≥ 0.9 have viral loads ≥ 200 copies/ml compared to 5.6% of those who live in areas with SVI < 0.9.

**Figure:**
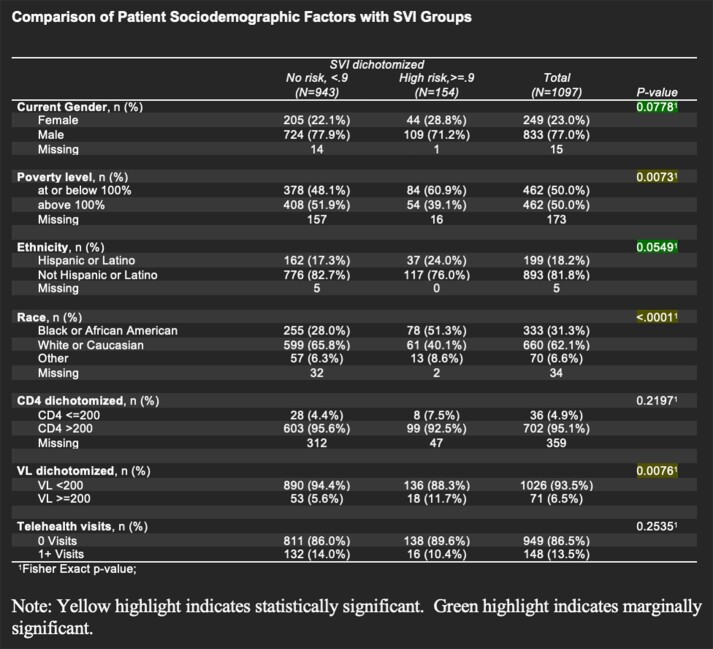

Map of SVI Score by Zip Code in Omaha, NE

**Figure:**
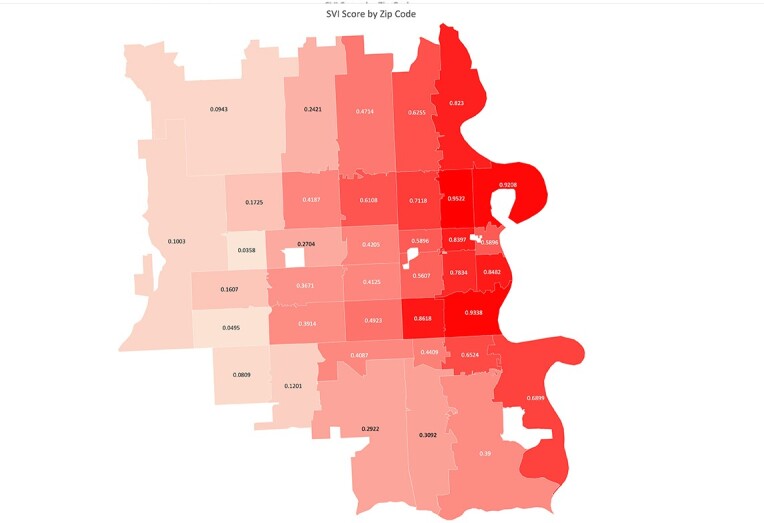

**Conclusion:**

The results are consistent with the study hypotheses that racial minority patients are more likely to reside in areas with high SVI and that high SVI is associated with poverty and poorer clinical outcomes.

**Disclosures:**

**All Authors**: No reported disclosures

